# Estimating the Impact of Plasma HIV-1 RNA Reductions on Heterosexual HIV-1 Transmission Risk

**DOI:** 10.1371/journal.pone.0012598

**Published:** 2010-09-13

**Authors:** Jairam R. Lingappa, James P. Hughes, Richard S. Wang, Jared M. Baeten, Connie Celum, Glenda E. Gray, Wendy S. Stevens, Deborah Donnell, Mary S. Campbell, Carey Farquhar, M. Essex, James I. Mullins, Robert W. Coombs, Helen Rees, Lawrence Corey, Anna Wald, for the Partners in Prevention HSV/HIV Transmission Study Team

**Affiliations:** 1 Department of Global Health, University of Washington, Seattle, Washington, United States of America; 2 Department of Medicine, University of Washington, Seattle, Washington, United States of America; 3 Department of Pediatrics, University of Washington, Seattle, Washington, United States of America; 4 Department of Biostatistics, University of Washington, Seattle, Washington, United States of America; 5 Department of Epidemiology, University of Washington, Seattle, Washington, United States of America; 6 Perinatal HIV Research Unit, University of the Witwatersrand, Johannesburg, Republic of South Africa; 7 Department of Molecular Medicine and Haematology, University of the Witwatersrand, Johannesburg, Republic of South Africa; 8 National Health Laboratory Service, University of the Witwatersrand, Johannesburg, Republic of South Africa; 9 Statistical Center for HIV/AIDS, Fred Hutchinson Cancer Research Center, Seattle, Washington, United States of America; 10 Vaccine and Infectious Disease Division, Fred Hutchinson Cancer Research Center, Seattle, Washington, United States of America; 11 Department of Immunology and Infectious Diseases, School of Public Health, Harvard University, Boston, Massachusetts, United States of America; 12 Department of Microbiology, University of Washington, Seattle, Washington, United States of America; 13 Department of Laboratory Medicine, University of Washington, Seattle, Washington, United States of America; 14 Reproductive Health and HIV Research Unit, University of the Witwatersrand, Johannesburg, Republic of South Africa; University of Cape Town, South Africa

## Abstract

**Background:**

The risk of sexual transmission of HIV-1 is strongly associated with the level of HIV-1 RNA in plasma making reduction in HIV-1 plasma levels an important target for HIV-1 prevention interventions. A quantitative understanding of the relationship of plasma HIV-1 RNA and HIV-1 transmission risk could help predict the impact of candidate HIV-1 prevention interventions that operate by reducing plasma HIV-1 levels, such as antiretroviral therapy (ART), therapeutic vaccines, and other non-ART interventions.

**Methodology/Principal Findings:**

We use prospective data collected from 2004 to 2008 in East and Southern African HIV-1 serodiscordant couples to model the relationship of plasma HIV-1 RNA levels and heterosexual transmission risk with confirmation of HIV-1 transmission events by HIV-1 sequencing. The model is based on follow-up of 3381 HIV-1 serodiscordant couples over 5017 person-years encompassing 108 genetically-linked HIV-1 transmission events. HIV-1 transmission risk was 2.27 per 100 person-years with a log-linear relationship to log_10_ plasma HIV-1 RNA. The model predicts that a decrease in average plasma HIV-1 RNA of 0.74 log_10_ copies/mL (95% CI 0.60 to 0.97) reduces heterosexual transmission risk by 50%, regardless of the average starting plasma HIV-1 level in the population and independent of other HIV-1-related population characteristics. In a simulated population with a similar plasma HIV-1 RNA distribution the model estimates that 90% of overall HIV-1 infections averted by a 0.74 copies/mL reduction in plasma HIV-1 RNA could be achieved by targeting this reduction to the 58% of the cohort with plasma HIV-1 levels ≥4 log_10_ copies/mL.

**Conclusions/Significance:**

This log-linear model of plasma HIV-1 levels and risk of sexual HIV-1 transmission may help estimate the impact on HIV-1 transmission and infections averted from candidate interventions that reduce plasma HIV-1 RNA levels.

## Introduction

The risk of sexual transmission of HIV-1 correlates strongly with plasma HIV-1 level.[1], [2] This association has motivated proposed interventions (such as use of antiretroviral therapy (ART),[3], [4] therapeutic HIV-1 vaccines,[5] and treatment for co-infections[6]–[8] that reduce HIV-1 infectiousness by reducing levels of plasma HIV-1 RNA. For example, epidemiologic studies have found that reactivation of herpes simplex virus type 2 (HSV-2) is associated with increased plasma and genital HIV-1 concentrations, and short-term clinical trials have demonstrated that HSV-2 suppressive therapy (using acyclovir or valacyclovir) significantly reduces plasma HIV-1 levels.[9]–[13] Based on these findings, we conducted the Partners in Prevention HSV/HIV Transmission Study to determine whether HSV-2 suppression in an HIV-1/HSV-2 dually infected person reduced HIV-1 transmission to the seronegative partner among African HIV-1 serodiscordant heterosexual couples (one partner HIV-1 infected and the other uninfected). The primary analysis of this trial found that, despite a 73% reduction in genital ulcer disease due to HSV-2 and a 0.25 log_10_ reduction in plasma HIV-1 RNA levels compared to the placebo arm, HSV-2 suppression with acyclovir did not prevent HIV-1 transmission (hazard ratio 0.92, 95% CI (0.60–1.41), p = 0.70).[14] Although acyclovir reduced plasma HIV-1 RNA levels by a similar magnitude as previous controlled trials,[9]–[13] the effect was not sufficient to significantly reduce HIV-1 transmission risk.

This finding raises the question of what reduction in plasma HIV-1 levels would be required to reduce HIV-1 heterosexual transmission risk. Detailed characterization of the relationship of plasma HIV-1 RNA to HIV-1 infectiousness could help evaluate the impact of proposed HIV-1 prevention interventions that operate by reducing plasma HIV-1 RNA. We used the prospectively collected data from this clinical trial to model the relationship between plasma HIV-1 RNA in HIV-1 infected partners and HIV-1 transmission risk in this population.

## Methods

The clinical trial study design, including participant screening, enrollment, follow-up, and laboratory methods, has been described in detail elsewhere.[14] Briefly, stable heterosexual HIV-1 serodiscordant couples were recruited in 14 sites in East and southern Africa. The HIV-1 infected partner, dually-infected with HSV-2 and with a CD4 count ≥250 cells/mm3, was randomized to HSV-2 suppression (acyclovir 400 mg orally twice daily) or placebo, with study drug provided at monthly visits. HIV-1 serostatus in the initially HIV-1 uninfected partner was assessed quarterly for up to 24 months by HIV-1 rapid assay, with HIV-1 seroconversions confirmed by ELISA and Western blot. HIV-1 transmission events were classified as “linked” (i.e., transmission from an HIV-1 infected individual to his/her enrolled partner) by comparing HIV-1 *env* and/or *gag* sequences of viruses from plasma of the HIV-1 seroconverters and the HIV-1 infected partner with whom they enrolled in the study.[14], [15] End of study central laboratory testing demonstrated that 27 of the 3408 couples did not meet the HIV-1 (n = 3) or HSV-2 (n = 24) serologic eligibility criteria leaving data from 3381 couples for this analysis. The HIV-1 transmission endpoint for this analysis was genetically-linked HIV-1 seroconversion including 17 linked seroconversions identified at the first quarterly study visit and retrospectively found to be HIV-1 RNA RT-PCR positive at enrollment (and therefore not included in the previously reported primary study analysis[14]).

Plasma HIV-1 RNA measurements (COBAS AmpliPrep/COBAS TaqMan HIV-1 RNA assay, version 1.0, Roche Diagnostics, Indianapolis, IN with a limit of quantification of 240 copies/mL) were performed at the University of Washington on acid-citrate dextrose plasma collected from the HIV-1 infected partner at the 0, 3, 6, and 12-month visits and at the final study visit. Since plasma RNA levels from HIV-1 infected individuals in this population were highly correlated over time (intraclass correlation  = 0.69), visits without measured plasma HIV-1 RNA level, but where HIV-1 seroconversion in the partner was evaluated, were assigned the last measured plasma HIV-1 RNA level carried forward. Undetectable plasma HIV-1 RNA levels were included if the participant was known to be taking ART or if plasma HIV-1 RNA was undetectable at a previous or subsequent visit, thereby reducing the likelihood that the undetectable value was a laboratory error. When included in this analysis, undetectable RNA levels were set equal to half the limit of quantification (120 copies/mL). For all seroconverters, plasma HIV-1 RNA was evaluated at the visit prior to HIV-1 seroconversion.

Data from both randomization arms of this clinical trial were used to model the relationship between plasma HIV-1 RNA level and risk of linked HIV-1 seroconversion. The (natural) log of HIV-1 transmission risk was modeled as a linear function of log_10_ plasma HIV-1 RNA using a Cox model with time-varying plasma HIV-1 RNA level. A more flexible model for log_10_ plasma HIV-1 RNA that used a natural cubic spline with 4 knots was also evaluated, and based on the results (see below) a linear model was selected for subsequent analysis. Since risk of transmission is commonly depicted on the non-log scale, this format was used to graph this relationship.

Since 36 linked transmission events had plasma HIV-1 measured at a visit prior to documented seroconversion, we used a sensitivity analysis to evaluate the use of the first positive HIV-1 test by either serology or RT-PCR as the endpoint in the Cox model. We evaluated the following co-factors for HIV-1 transmission in this cohort for interaction with or confounding of the relationship between plasma HIV-1 RNA level and HIV-1 transmission: baseline HSV-2 serology in the initially HIV-1 uninfected partner, male circumcision in HIV-1 infected and HIV-1 uninfected partners, and gender. We also assessed unprotected sex reported by the initially HIV-1 uninfected partner a time-dependent factor measured every three months.

We developed a predictive tool to estimate the overall reduction in HIV-1 transmissions for a given reduction in plasma HIV-1 RNA concentrations and, when applied to a hypothetical population, to estimate the impact of such reductions in plasma HIV-1 RNA on HIV-1 infections averted. We assume that the HIV-1 incidence rate and plasma viral RNA distribution in the hypothetical population is stable. To illustrate the use of this tool, a fixed plasma HIV-1 RNA reduction was applied to two HIV-1 infected populations defined by the groups of the 4 highest versus 10 lowest HIV-1 incidence study sites. We then calculated the number of HIV-1 infections averted over one year as the difference between the expected HIV-1 infections in a population of 100,000 seronegative persons exposed to HIV-1 infected partners with the indicated population distribution of plasma HIV-1 RNA.

The clinical trial protocol, including use of clinical trial data for studies of determinants of HIV-1 transmission, and informed consent documents were reviewed and approved by human subjects research committees at the University of Washington and all local study site and affiliated institutions. All clinical trial participants provided written informed consent for participating in this research. The trial is registered through ClinicalTrials.gov as NCT00194519 [http://clinicaltrials.gov/ct2/show/NCT00194519?term=NCT00194519&rank=1].

## Results

Among the 3381 couples included in this analysis, the HIV-1 infected partner was female in 2284 and male in 1097 couples. The median CD4 count was 462 cells/mm3 (Table 1). A total of 5017 persons-years of follow-up were accumulated, of which 4756 (94.7%) had plasma HIV-1 RNA data available. During this follow-up, we observed 108 linked HIV-1 transmissions (incidence 2.27 per 100 person-years).

**Table 1:** Demographic characteristics of the study population (N = 3381).

Baseline Characteristic	Number (%) or Median (IQR)
**HIV-1 infected partner**	
Women	2284 (68%)
Median CD4 count (cells/mm^3^)	462 (347–631)
**HIV-1 uninfected partner**	
HSV-2 seropositive	2294 (68%)
**Couple characteristics**	
Median number of sex acts in the month prior to enrollment	4 (2–8)
Couples reporting unprotected sex acts in the month prior to enrollment	1252 (37%)

### Model characteristics

**Figure 1:**
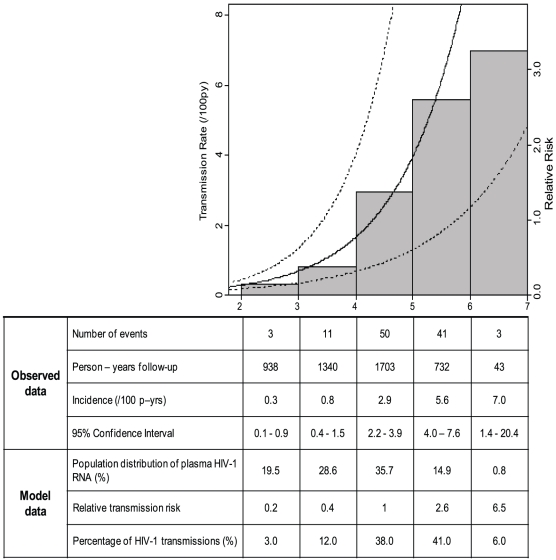
The left y-axis is scaled so that a RR of 1 corresponds to the observed overall risk of transmission in the cohort (2.27% per person-year) at the median (log) viral load. The model assumes a linear relationship between log risk of HIV-1 transmission and log_10_ plasma HIV-1 RNA level with the solid line as the model-predicted risk of transmission and dashed lines are 95% point-wise confidence intervals (p<0.0001). The numbers on the x-axis for the graph (plasma HIV-1 RNA) also indicate the range of plasma HIV-1 RNA values for each column of the table. The observed data (number of HIV-1 transmission events, person-years of follow-up, calculated HIV-1 incidence and 95% confidence interval [CI]) and model data (study population distribution of plasma HIV-1 RNA levels and proportion of transmissions) by level of plasma HIV-1 RNA are provided below the graph. The 0.5% of the population with plasma HIV-1 RNA level <2 log_10_ copies/ml and no observed transmissions are not included in this graph.

We fitted a log-linear relationship between log_10_ plasma HIV-1 RNA and HIV-1 transmission (Figure 1; p<0.0001). The model generally fitted closely with measured incidence. At the highest plasma virus levels (6–7 log_10_ copies/mL) both observed linked HIV-1 transmissions (n = 3) and observed follow-up (43 person-years) were low, and the observed risk was flatter than the predicted risk curve; however, a more flexible cubic spline model did not significantly improve the fit in this range (p = 0.21).

During the 938 person-years of follow-up associated with an HIV-1 infected partner having low plasma HIV-1 RNA (2 to 3 log_10_ copies/mL), three HIV-1 transmissions were identified. The maximum plasma HIV-1 RNA level measured at any point during study follow-up for these three HIV-1 transmitting partners were 3.19, 3.95 and 4.71 log_10_ copies/mL.

The model predicts that, given the population distribution of HIV-1 plasma RNA in this study, at plasma HIV-1 levels between 5–6 log_10_ copies/mL the risk of HIV-1 transmission is 2.6-fold greater than at 4–5 log_10_ copies/mL, 6.5-fold greater than at 3–4 log_10_ copies/mL and 16.6-fold greater than at 2–3 log_10_ copies/mL. Since these data fit a log-linear relationship, each one-log increase in plasma RNA results in a 2.49-fold increased risk of transmission. More specifically, reductions in plasma HIV-1 of 0.5, 0.74 and 1.48 log_10_ copies/mL applied to *any* plasma HIV-1 level will result in 37%, 50% and 75% reductions in HIV-1 transmission risk, respectively (Table 2). In a sensitivity analysis using time to first indication of HIV-1 infection (by earliest of serology or RT-PCR) instead of time to HIV-1 seroconversion, the plasma HIV-1 reduction required to decrease transmission risk by 50% was not meaningfully changed (0.82 versus 0.74 log_10_ copies/mL).

**Table 2:** Plasma HIV-1 RNA reductions (and 95% Confidence Intervals [CI]) predicted to result in selected levels of HIV-1 transmission risk reduction.

HIV-1 transmission risk reduction	Plasma HIV-1 RNA reduction (log_10_ copies/mL)	95% CI (log_10_ copies/mL)
25%	0.31	0.25–0.40
33%	0.43	0.34–0.56
37%	0.50	0.40–0.65
50%	0.74	0.60–0.97
67%	1.18	0.95–1.56
75%	1.48	1.19–1.95

We assessed whether the HIV-1 transmission risk versus plasma RNA relationship differed based on HIV-1 incidence rates. We applied the model to the subset of data from study sites with the highest HIV-1 transmission rate (average 5.8 per 100 person-years) versus study sites with lower HIV-1 incidence (average 1.8 per 100 person-years) and found no meaningful difference in the log hazard ratio of transmission per log change in plasma HIV-1 level. Furthermore, factors known to affect HIV-1 transmission (history of any unprotected sex, gender, HSV-2 seropositivity, male circumcision of the HIV-1 uninfected partner) did not significantly interact with or have a confounding effect on the relationship between plasma HIV-1 RNA and risk of transmission. Thus, changes in these characteristics in a population will not significantly affect the impact of changes in plasma HIV-1 RNA level on HIV-1 infections averted making this model broadly applicable for this purpose.

### Population-specific impact of plasma HIV-1 reductions on HIV-1 infections averted

Our log-linear model predicts that the *proportional* decrease in HIV-1 transmission risk associated with a given reduction in log_10_ plasma HIV-1 RNA is independent of the baseline plasma HIV-1 level. However, the *absolute* reduction in transmission risk associated with a given reduction in plasma HIV-1 RNA will, of course, be greater at higher plasma HIV-1 levels. Thus, the number of HIV-1 infections averted by a reduction in plasma HIV-1 will tend to be greater at higher plasma virus levels. To illustrate this, we calculated the impact of a 0.74 log_10_ reduction in plasma HIV-1 RNA applied to a population with the plasma HIV-1 distribution of the high HIV-1 incidence versus the low-incidence subset of clinical trial sites. This plasma HIV-1 RNA reduction would translate to 2903 HIV-1 infections averted per 100,000 HIV-1 uninfected persons in the high-incidence population compared to 902 per 100,000 in the low-incidence population (constituting 50% of the new HIV-1 infections expected from each of these populations, respectively) (Table 3).

**Table 3:** Impact of plasma HIV-1 RNA reduction on HIV-1 incidence and infections averted in high versus low incidence cohorts.

Cohort Characteristics	Plasma HIV-1 RNA reduction (log_10_ copies/mL)	Plasma HIV-1 RNA Level						
		≤2	>2 to 3<3	>3 to 4	>4 to 5<5	>5 to 6	>6 to 7	Total
**High incidence population (HIV-1 incidence - 5.8%/year)** 1								
Population distribution of plasma HIV-1 RNA (%)	n/a	0.1	17	24.8	40.3	16.5	1.3	100
Percentage of HIV-1 transmissions (%)	n/a	<0.01	3	9	39	41	8	1.00
# HIV-1 Infections averted (/year)^2^	0.74	0	73	274	1136	1186	234	2903
**Low incidence population (HIV-1 incidence - 1.8%/year)** 1								
Population distribution of plasma HIV-1 RNA (%)	n/a	0.2	20.2	29.1	35.0	14.7	0.8	100
Percentage of HIV-1 transmissions (%)	n/a	<0.01	3	12	38	41	6	1.00
# HIV-1 Infections averted (/year)^2^	0.74	0	30	112	343	367	50	902

1 High and low incidence populations include the subset of clinical trial sites with the highest and lowest HIV-1 incidence, respectively.

2 The log-linear model is used to estimate HIV-1 infections averted per year as the difference in HIV-1 infections that would occur in that plasma RNA stratum if the plasma HIV-1 RNA reduction is applied to a population of 100,000 HIV-1 uninfected persons compared to the number of HIV-1 infections expected in that population in the absence of the plasma HIV-1 RNA reduction. The total number of new HIV-1 infections expected per year in these hypothetical examples are 5800 and 1800 for the high and low incidence populations, respectively.

Given the increased risk of transmission at higher levels of plasma HIV-1 RNA, we estimated the potential impact on HIV-1 infections averted in the high incidence population if HIV-1 infected individuals were targeted with a reduction in plasma HIV-1 levels based on their baseline plasma HIV-1 level. We found that an intervention that reduced plasma HIV-1 by 0.74 log_10_ delivered to the 16.5% of individuals with plasma HIV-1 RNA between 5–6 log_10_ copies/mL in the high incidence population would avert 1186 HIV-1 infections, compared to 1136 infections averted for the same plasma HIV-1 RNA reduction applied to the 40.3% of persons in that population with plasma HIV-1 RNA between 4–5 log_10_ copies/mL. Overall, in this example, 88% of the total HIV-1 infections averted were derived from the 58% of the population with plasma HIV-1 RNA levels ≥4 log_10_ copies/mL. We have included in Table S1 an application of this model that calculates HIV-1 transmission risk and HIV-1 infections averted from data input by a user for the plasma HIV-1 distribution and HIV-1 transmission incidence in their selected study population.

## Discussion

The Partners in Prevention HSV/HIV Transmission Study provides the largest and most detailed database to date for modeling the relationship of plasma HIV-1 RNA and risk of heterosexual HIV-1 transmission, allowing us to model a predicted impact of a reduction in plasma HIV-1 RNA on HIV-1 infectiousness. Our model assumes a linear relationship between the logarithm of the risk of HIV-1 transmission and the logarithm of plasma HIV-1 RNA; this assumption is validated by the observed data particularly in the range of 3 to 6 log_10_ plasma RNA. Thus, plasma HIV-1 RNA reductions of 0.5 log_10_ and 0.74 log_10_ copies/mL are associated with 37% and 50% reductions in the risk of HIV-1 transmission, respectively, regardless of the baseline level of plasma HIV-1 RNA. Our findings are in close agreement with those from a HIV-1 serodiscordant couples cohort in Rakai, Uganda for which 0.5 and 0.74 log_10_ plasma HIV-1 RNA reductions were associated with 36% and 48% reductions in HIV-1 transmission, respectively (reference (2); and personal communication, Dr. Laith Abu-Raddad, Weill Cornell Medical College, Qatar). However, it should be noted that viral sequence-based linkage confirmation performed in our study cohort was not performed in the Rakai study. Linkage confirmation was performed in a prior study of Zambian HIV-1 serodiscordant couples [1], but the specific measures of the relationship of plasma HIV-1 and transmission risk discussed here have not been reported for that cohort. Our findings are also similar to other recent studies reporting reductions in HIV-1 transmission risk of 26%[16] and 40%[17] in association with a 0.5 log_10_ copies/mL plasma HIV-1 RNA decrease. In addition to incorporating genetic linkage of transmissions, our model is based on a larger cohort of HIV-1 serodiscordant couples, with longer duration of follow-up, than previous studies.

Epidemiologic co-factors for HIV-1 transmission (gender, HSV-2 infection in the HIV-1 uninfected partner, male circumcision in the HIV-1 uninfected partner and history of any unprotected sex) did not interact with or confound the relationship of plasma HIV-1 RNA levels to HIV-1 transmission risk. Since, based on study eligibility criteria, all HIV-1 infected partners in this study cohort were dually infected with HSV-2, we could not assess HSV-2 infection in the HIV-1 infected partner for interaction with the relationship of HIV-1 plasma level and HIV-1 transmission. However, given the close agreement between the model derived from this cohort compared to that derived from the Rakai cohort, where the prevalence of HSV-2 antibody among HIV-1 infected persons was lower (67%),[18] it is unlikely that HSV-2 infection strongly interacts with this relationship. Therefore, we infer that our model is both accurate and generally applicable to populations with varied characteristics, particularly within the 2 to 6 log_10_ copies/mL range of plasma HIV-1 levels where this model has greatest power.

Our data suggest that HIV-1 transmission risk increases substantially with no evidence of biological saturation through a range of plasma HIV-1 levels from 3 to 6 log_10_ copies/mL. In the range of 6–7 log_10_ copies/mL we had a small number of events (n = 3) with few person-years of follow-up (n = 43), so we cannot be certain that the log-linear relationship holds at such high levels of plasma HIV-1 RNA. Increased power to ascertain HIV-1 transmission risk in this high plasma HIV-1 stratum would necessitate targeting HIV-1 serodiscordant couples with one acutely infected and one HIV-1 uninfected partner, who are difficult to identify. At the low end of the plasma HIV-1 RNA spectrum (HIV-1 infected partners with plasma HIV-1 levels <3 log_10_ copies/mL), we only observed three HIV-1 transmission events over 968 person-years of follow-up. Given the small number of events, it is possible that, in some cases, HIV-1 levels immediately prior to HIV-1 transmission were transiently higher than those documented at their scheduled visits. More generally, since plasma HIV-1 levels were measured at 0, 3, 6, 12 months and at study exit, and not at every quarterly visit, our estimate of the relationship between HIV-1 transmission risk and HIV-1 plasma viral load may be somewhat attenuated. However, given the high correlation of plasma HIV-1 RNA levels over time in our dataset, our model is likely robust.

Antiretroviral therapy is currently the only therapeutic intervention that consistently reduces plasma HIV-1 RNA by more than 0.7 log copies/mL.[19], [20] A recent analysis of our cohort documented that ART use reduced risk of HIV-1 transmission by 92% (p = 0.004) and an ongoing randomized trial will assess the long-term effect of ART on HIV-1 transmission[21]. However, current resources constrain many countries from providing ART to all HIV-1 infected persons meeting current international guidelines for treatment, and recent proposals[4] to perform HIV-1 testing more broadly (“test and treat”) will accentuate this challenge.

This model demonstrates that a 0.74 log_10_ copies/mL reduction in HIV-1 plasma RNA reduces HIV-1 transmission by 50%, and that 90% of these infections averted could be achieved by targeting the plasma HIV-1 RNA reduction to a subset of the population with plasma HIV-1 RNA levels ≥4 log_10_ copies/mL (less than 60% of HIV-1 infected persons, across a range of scenarios). While ART for HIV-1 prevention may be made more cost-effective by targeting persons with plasma HIV-1 RNA ≥4 log_10_ copies/mL, given the current high cost and limited availability of plasma HIV-1 RNA monitoring, significant advances in this technology would be required before this approach could be implemented in ‘treatment for prevention’ algorithms[22] in resource-limited settings. In the absence of a low-cost HIV-1 RNA assay, the model and tool we have developed could be used to inform public health resource allocation or to prioritize populations considered for HIV-1 prevention clinical trials.

This model does not explicitly include the impact of stage of HIV-1 infection on HIV-1 transmission potential. Given the long duration of time that individuals with clinically latent HIV-1 infection can remain infectious, their overall contribution to HIV-1 transmission may be higher than individuals at early and advanced HIV-1 disease stages associated with higher plasma HIV-1 RNA levels.[23] However, the plasma HIV-1 RNA levels contributing to most HIV-1 infections in our study population (i.e., ≥4 log_10_ copies/mL) encompasses the median plasma HIV-1 level of clinically latent infections potentially mitigating the impact of explicit inclusion of stage of HIV-1 infection in the model.[24] This model also does not include additional behavioral factors, or viral and host factors that also contribute to HIV-1 infectiousness. However, by modeling the relationship of plasma HIV-1 RNA level to HIV-1 transmission risk, this analysis captures the principal determinant of that risk. We are also in the process of evaluating a per sexual contact model to address differences in sexual frequency and condom use. Finally, our evaluation of HIV-1 infections averted provides an approximate estimate derived by extrapolation from the stable partnerships followed through the clinical trial. More complex models are required to incorporate population-level estimations with multiple risk groups having different saturation levels and the impact of onward transmission.

Non-ART interventions, such as treatment of co-infections that are associated with increased plasma HIV-1 levels, are currently being evaluated to reduce HIV-1 infectiousness and disease progression. If such interventions reduce plasma HIV-1 levels by >0.7 log_10_ copies/mL, they may also provide a valuable tool for prevention of HIV-1 transmission. HSV-2 suppression with standard doses of acyclovir (as assessed in the Partners in Prevention HSV/HIV Transmission Study) did not meet this goal, and ongoing studies are evaluating whether higher doses of herpes suppression are associated with a greater reduction in plasma HIV-1 levels. Given the costs and challenges of trials evaluating interventions to reduce HIV-1 transmission, there is a need to prioritize which candidate interventions are evaluated. This model of plasma HIV-1 RNA levels and HIV-1 transmission risk provides a useful assessment tool for estimating the potential HIV-1 prevention impact of reductions in plasma HIV-1 levels.

## Supporting Information

Table S1 (0.02 MB XLS)
